# Mr. Key's Cases of Cow-Pock Failure

**Published:** 1808-07

**Authors:** Thomas Key

**Affiliations:** Fenchurch Street


### *To the Editors of the Medical and Phyfical Journal.*

GENTLEMEN,

ASSOCIATED as vaccination is with the best interests of society, I cannot conceive a greater delinquent than the Practitioner, who by concealment, or a fraudulent statement of any case, imposes upon the public; it must, if persevered in, prove the greatest blessing, or the severest scourge, hitherto promulgated; notwithstanding, therefore, the the evidence in its favour is so respectable, the importance connected with its adoption, requires that no unfavourable evidence, if substantiated, should be rejected. Influenced by such sentiments, I am induced to circulate for general information, three cases of cow-pock failure as occurring in one family.

Julia Rose, daughter of Mr. Rose, High Street, Southwark, when six months old, was vaccinated by myself, and I have with my own, the evidence of Dr. Denman, as to the correctness of the vaccine vesicle. Frederic and Josephine Rose were vaccinated by a very respectable Practitioner; the first when five, the latter when one week old. Julia, when she had the small-pox, was seven years old; Frederic five, and Josephine three. That there should be no question as to the eruptions upon Julia being variolous, I took some matter from one of the pustules and inoculated an infant five months old, to which it communicated a mild small-pox. Frederic and Josephine received the disease casually from their sister, and, like her, had a mild small-pox. It has, I believe, in the few instances of failure hitherto recorded, been admitted, that where vaccination has failed in giving complete protection, it has usually insured a mild small-pox. There is strong presumptive evidence in favour of that opinion in the case of Julia Rose, who, when first indisposed, was suspected of sickening for the measles, and every aid by warm drinks, warm clothing, and a close room, employed to throw out the eruption; under such treatment therefore it may reasonably be inferred, that she could not have escaped a confluent disease, had not the previous vaccination modified the susceptibility of the constitution to receive the small-pox.

Let it not be understood that I possess a sentiment hostile to vaccination; for, admitting the case I have related (as my own) to be an instance of positive failure, it is the only one in an extensive practice of nine years, during which, I have invariably resisted variolous inoculation. My experience, therefore, by a comparison of variolous with vaccine inoculation, is decidedly in favour of the latter.

While on the subject of vaccination, it may not be irrelevant to remark, that either from a reluctance in Practitioners to admit of any imperfection in their practice, or from some cause interfering with their attention to the progress of the vaccine vesicle, I suspect there have been instances where the vaccination was not correct, yet suffered to pass without noticing the probable insecurity; in this observation observation I am supported by what occurred in a child inoculated by a gentleman in the County of Sussex.

It may not be convenient in an extensive practice, to register every individual case; but it is required of every Practitioner, when he sees an irregularity in the progress of the vaccine vesicle, to express his doubts as to the protection it may insure, to urge the necessity of a re-inoculation at some future period; nor would it to any great extent interfere with his engagements, were he to register such occurrences; by which, supposing small-pox to supervene, he might rescue vaccination from that unjust obloquy which has been attached to it.

Were Science the only object of those who go in pursuit of unsuccessful cases, the public would have to thank them, but some there are, who, it appears, only seek to impose, and the illiberality that accompanies their inquiries serves to detect them.

It is obvious that I am the decided friend of vaccination, but I am at the same time desirous that it should pass the ordeal of a free and rational inquiry; I must therefore deprecate that conduct which affects to support its infallibility, by refusing to admit the possibility of its failure. Cow-pock inoculation requires no such aid, and the public will be inclined to suspect what appears to require fraud for its protection should cease to be practised.

THOMAS KEY.

*Fenchurch Street, June,* 18, 1808.

